# A Third Type of Defensive Behavior in the Tenebrionid Beetle *Zophobas atratus* Pupae

**DOI:** 10.1673/031.013.3301

**Published:** 2013-04-18

**Authors:** Toshio Ichikawa, Hirofumi Sakamoto

**Affiliations:** 1 Department of Biology, Faculty of Sciences, Kyushu University, Fukuoka 812-8581, Japan; 2 Basic Life Science, Graduate School of System Life Sciences, Kyushu University, Fukuoka 812-8581, Japan

**Keywords:** abdominal rotation, cannibalism, mechanosensilla, predators, parasitoids

## Abstract

Pupae of the tenebrionid beetle *Zophobas atratus* Fabricius (Coleoptera: Tenebrionidae) exhibit two types of reflex abdominal motions in response to tactile stimulation: circular rotation and lateral bending to close pinching devices (gin-traps). In the present study, the pupa exhibited novel, sequential abdominal movements at 0.3–2.2 sec after the onset of mechanical stimulation. The most effective stimulation was gentle, double brushing on the ventral surface of an abdominal segment (sternite). The sequential abdominal movements consisted of the following three types of discrete elementary motions (100–350 ms in duration): rapid vibration of 30–40 Hz, circular rotation (or swing), and small wiggling movements. A sequence of abdominal movements generally started with a few bouts of vibration, but the number and order of subsequent motions varied considerably among different sessions and conditions. A restrained pupa often showed a prolonged sequence of many motions, including several rotations, whereas an unrestrained pupa often shortened the sequence by skipping a few rotations after the displacement of its whole body induced by the first abdominal rotation. Stimulation of two types of mechanosensitive sensilla, the hair sensilla (touch sensors) and campaniform sensilla (strain sensors), seemed to be necessary to initiate the defensive response. In natural environments, crawling of a small predator (or parasitoid) on the surface of the abdomen or repeated attacks of a large predator may induce this defensive response in the pupae.

## Introduction

The pupal stage of a holometabolous insect is a metamorphic period during which most larval tissues are replaced with newly formed imaginai tissues. However, many pupae retain larval abdominal muscles that allow them to move the abdomen vigorously in response to mechanical stimulation. Abdominal movement serves a defensive function in the pupae of a few coleopteran and lepidopteran families that have sclerotized jaws at the dorsal or lateral margins of jointed abdominal segments (Hinton 1946, 1955; Bouchard and Steiner 2004). The jaws are rapidly closed by contractions of the abdominal intersegmental muscles and form simple traps (gin-traps) capable of pinching small potential predators. The defensive function of pinching was examined in the pupae of the hawkmoth, *Sphinx ligustri* (Bate 1973a, b, c), the tenebrionid beetle *Tenebrio molitor* (Hinton 1946; Wilson 1971), and the ladybird beetle, *Cycloneda sanguinea* (Eisner and Eisner 1992). The pupae of *T. molitor* exhibit another type of defensive response: a rapid circular rotation of the abdomen, fringed with many spines, against tactile stimulation of their free appendages (Hollis 1963; Askew and Kurtz 1974). This response may be effective in protecting the pupae from larval cannibalism (Ichikawa and Kurauchi 2009).

Others have observed mechanosensory mechanisms and trajectory patterns of abdominal movements in the pupae of a largesized tenebrionid beetle, *Zophobas atratus* Fabricius (Coleoptera: Tenebrionidae) (Ichikawa et al. 2012a, b; Kurauchi et al. 2011). Both types of abdominal movements result from stimulation of campaniform sensilla (CS) (strain sensors) in the pupal cuticle, although many sensory hairs (touch sensors) are on the surface of almost all parts of the body (Kurauchi et al. 2011). The rotation of the abdomen occurs in an all-or-none fashion, and all abdominal segments appear to be involved in this response (Ichikawa et al. 2012b). In contrast, the closing of the gin-trap is graded, and different numbers of abdominal segments appear to bend the abdomen laterally or move the abdomen circularly (Ichikawa et al. 2012a).

In the present study, *Z. atratus* pupae exhibited a novel defensive behavior that was not a single reflex to a tactile stimulus but a series of abdominal movements. These movements consisted of multiple elementary motions that occurred after activating the pupae by brushing a specific region of pupal body or by strongly stimulating the pupa to induce many reflex responses.

## Methods and Materials

### Animals and preparations

The mature larvae of giant mealworms *Z. atratus* were purchased from a local supplier. The larvae were maintained under crowded conditions in a mixture of peat moss and sawdust and were fed fresh Japanese radishes. Individual larvae were isolated in a plastic cup for pupation. The pupae were maintained at 26 ± 1° C under a 16:8 L:D photoperiod for an average pupal period of 13 days.

Day 0 pupae (6–24 hr after pupation), unless otherwise noted, were usually used for the analysis of abdominal movements under restrained or unrestrained conditions. In the restrained condition, the dorsal part of the thorax of the pupae were fixed to a platform with melted paraffin and maintained at 25–26° C. In the unrestrained condition, the pupae were laid on their dorsal or lateral sides on a flat substrate covered with a sheet of sandpaper. All pupae were maintained under undisturbed conditions for more than 2 hr before use.

### High-speed photography

A digital video camera (HAS-200R; Ditect, Tokyo, Japan) placed behind the pupa (abdomen) was used to view its behavior from the posterior direction. High-speed movies (200 frames/sec) were made and analyzed according to a method described in a previous paper (Ichikawa et al. 2012a).

### Recording of abdominal motions

Vibration and small motions in the pupal body were monitored using two devices. The first device was a mechanical sensor (strain gauge) connected to the side wall of a Styrofoam block that could transmit the vibration or movement of the pupal body. The second was an optical device that consisted of a laser pointer and a photocell. The laser beam was adjusted to pass the lateral edge of an abdominal segment, and the photocell was placed behind the segment. Calibrations of the mechanical and optical sensors indicated that outputs of the mechanical sensors were linearly related to the movements of the block, but those of the optical sensors were non-linearly related. Electrical signals from the sensors were amplified, digitized, and stored on a computer equipped with an analog/digital converter (1401 pulse; Cambridge Electronic Design, www.ced.co.uk).

### Mechanical stimulations

Responses to mechanical stimulation were usually induced by brushing the cuticular surface of a sensitive region of the pupal body by using a fine writing brush. Occasionally, a glass rod with a fire-polished, round tip (diameter 1.0 mm) was used to prod the region. When the brush was applied twice to a testing area, the interval between two successive brushings was 0.4–0.5 sec. For multiple stimulations, such double brushings were usually performed at an interval of 1 min. The stimulation was usually repeated 10 times to obtain mean response rate of a single pupa. Brushings were mostly done to a small ventral area 2–3 mm lateral to the midline of the fourth abdominal segment. Although the force of a manual brushing could not be controlled precisely, it was estimated using a calibrated strain gauge. The estimated forces of soft, weak, and strong brushings were 0.2–0.3, 0.6– 0.7, and 1.7–1.8 mN, respectively. To induce an abdominal rotation, we prodded an elytron with a nylon filament (bending force 0.6 mN).

### Experimental protocols

To determine whether the CS played a significant role in the initiation of the defensive response, the elytron was used as a testing site because it had many CS and lacked hair sensilla (HS). However, there was a possibility that a mechanical force might bend the elytron to contact the underlying hind wing bearing a few long and several short HS (Kurauchi et al. 2011). To block this contact, quick drying glue was poured under the elytron on one side, but not under the elytron on the other side, as a control. The double brushing test was performed on the outer surface of the elytra.

To examine the responsiveness of an excited (or sensitized) pupa, its appendage (elytron or leg) was prodded 20 times with a nylon filament at an interval of 1 sec. One or two single brushings were delivered to the ventral surface of the fourth abdominal segment 10 sec (0 min), 30, 60, and 120 min after the end of sensitization.

### Statistical analyses

Data were statistically analyzed by non-parametric Dunnett test or Steel-Dwass multiple comparison test. Correlation between response rates and intervals of two brushings was analyzed by Kendall rank correlation test.

**Movie 1.**
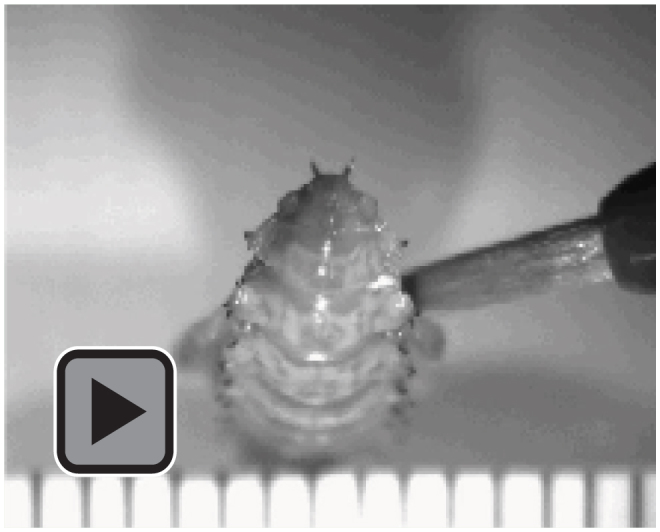
The response consists of six bouts of vibration, two rotations, and two wiggling motions. Click image to view video. Download video

## Results

When a *Z. atratus* pupa was violently pinched by fingers, it struggled to free itself by moving its abdomen vigorously. Just after release, the pupa often exhibited a series of complex abdominal movements. Similar movements often occurred following many defensive abdominal rotations evoked by repetitive (strong) tactile stimulation of the appendages. The pupa could be readily stimulated to perform such sequential abdominal movements, without the induction of any other reflex response, by a simple method of gently brushing the ventral surface of an abdominal segment (sternite) once or twice. In contrast, repeated prodding was not effective in evoking the response. The response profiles and movement patterns in restrained and unrestrained pupae were examined.

### Restrained pupae

**Figure 1.**
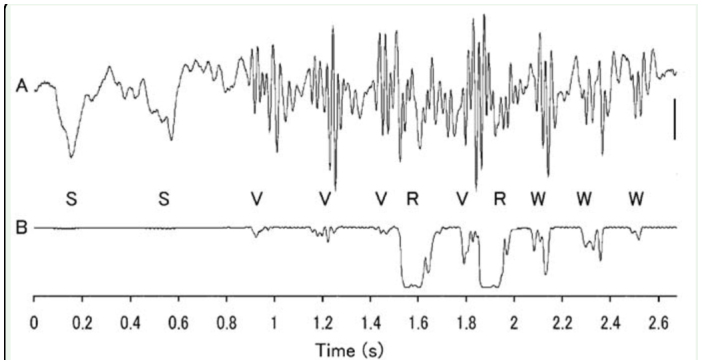
Sequence of abdominal movements as recorded on mechanical (A) and optical devices (B). Other letters represent artifact of weak stimulation (S), vibration (V), rotation (R), and wiggling (W). The scale is 0.05 mm (A) and an arbitrary unit (B). High quality figures are available online.

**Figure 2.**
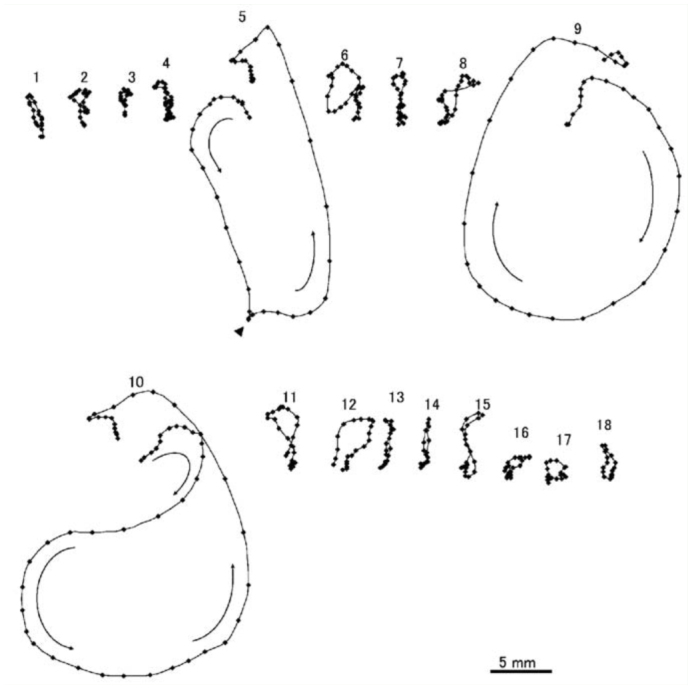
Trajectory pattern of prolonged sequence of movements due to stimulation, and recorded as elements 1 through 18. Vibration: elements 1–4, 6…8, and 11–15; rotation: 5, 9, and 10; small wiggling: 16–18. Start times of individual motions after the stimulation were 0.34 (1), 0.64 (2), 0.86 (3), 1.14 (4), 1.41 (5), 1.71 (6), 1.94 (7), 2.16 (8), 2.41 (9), 2.72 (10), 3.06 (11), 3.25 (12), 3.44 (13), 3.62 (14), 3.81 (15), 4.02 (16), 4.20 (17), and 4.41 (18) seconds. Arrows indicate the direction of rotations. The arrowhead indicates an abrupt change in the direction of movement. Note that an initial clockwise rotation of the tenth element changed to an anticlockwise rotation at the halfway point of the rotation. High quality figures are available online.

**Figure 3.**
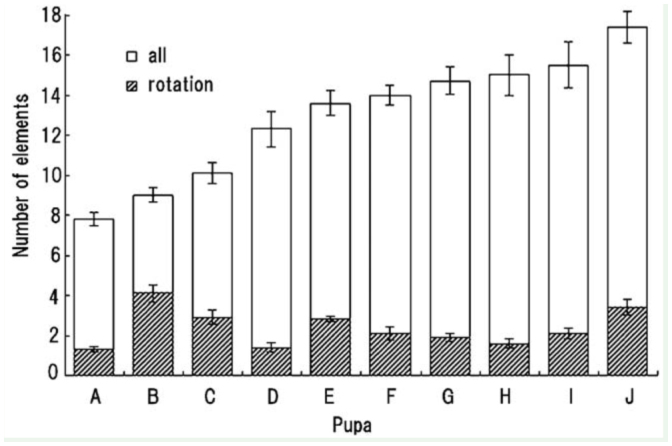
Variability in the number of elements involved in the behavioral response of pupae (A–J). Each bar indicates a mean value and standard error of the mean of 10 determinations. High quality figures are available online.

A double brushing was usually required to induce a sequence of abdominal movements in young pupae, and a single brushing could induce the response at a low probability of < 25%. The sequence of abdominal movements was usually initiated 0.3–2.2 sec after the onset of the second brushing and consisted of the following three types of elementary motions (duration, 100–350 ms): 3–5 cycles of vibration of all segments, a circular rotation (or swing), and a few small wiggling movements of several caudal segments (Movie 1). The movements typically started with a few or several bouts of vibration (frequency, 30–40 Hz; amplitude, 1.0–1.5 mm), followed by a few rotations and a few wiggling movements (Figure 1). However, the number and order of elementary motions were highly variable among different sessions, even in the same pupa. A short sequence (lasting 1–2 sec) had a few bouts of vibration, one rotation, and a few wiggling movements, while a prolonged sequence (lasting 4–5 sec) had up to 20 elementary motions, including multiple rotations (Figure 2). The first rotation usually moved the abdomen toward the stimulated side, and the directions of subsequent rotations were variable. The trajectory patterns of the rotations were not simple circular or elliptical, and an abrupt change in the direction or a reversal of the rotational direction often occurred halfway through the rotation (Figure 2). There was a large variation in the sequential abdominal movements among different pupae (Figure 3); less active pupae (e.g., pupa A in Figure 3) usually performed short sequential movements consisting of several elements, including one rotation, while very active pupae (e.g., pupae B and J) performed long sequential movements of more than three rotations (maximum, 8 rotations). Many other moderately active pupae performed a sequence of 12–16 elementary motions, including 2–3 rotations. The mean ± SEM delay in the responses was 0.91 ± 0.12 sec (50 determinations in 10 pupae), and the long delay responses were in contrast to the short delay rotation reflexes (0.051 ± 0.004 sec) determined using the same group of pupae.

**Figure 4.**
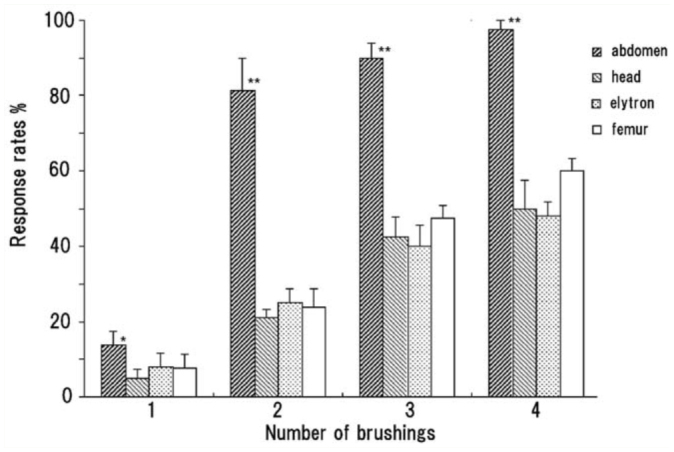
Percentage of pupae responding to weak brushings to different body parts. Each bar indicates a mean value and standard error of the mean from eight pupae. The abdomen significantly differs in the responsiveness from other three parts (**, *p* < 0.01; *, *p* < 0.05), while there are no significant differences among the three (Steel-Dwass multiple comparison test). High quality figures are available online.

Brushing the ventral surface of an abdominal segment was most effective in initiating an abdominal response, and only two or more successive brushings at an appropriate interval could initiate a delayed response at a probability of > 80% (Figure 4). Similar weak brushings applied to other body parts (e.g., the head (frons), ventral surface of the femur of the hind leg, and an elytron) could cause a response without any other reflex response; however, the response rates were usually lower than 60% (Figure 4). Stronger brushings of the appendages usually induced an abdominal rotation reflex. Brushing the dorsal surface of an abdominal segment evoked no response.

**Figure 5.**
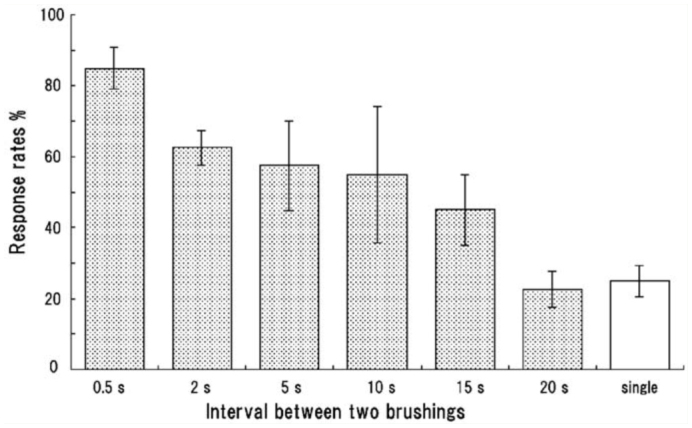
Percentage of pupae responding to two weak brushings at increasing time intervals. The first and second brushings were applied to the same ventral region of the fourth abdominal segment. Single indicates the first brushing only. Each bar indicates a mean value and standard error of the mean from five pupae. Response rates are inversely related to the intervals (τ = -0.76; *p* < 0.001) (Kendall rank correlation test). High quality figures are available online.

A double brushing at an appropriate interval appeared to be significant, and the relationship between the interval of brushings and responsiveness was examined. As shown in Figure 5, the response rate gradually decreased as the interval between the two brushings increased (or the second brushing was delayed). The rate was lowered to about 20%, the same level of a single brushing, when the second brushing was applied to the same region of the abdominal segment 20 sec after the first brushing. Similar results were obtained when two brushings were applied to different ventral regions of the same segment or different segments (data not shown).

**Figure 6.**
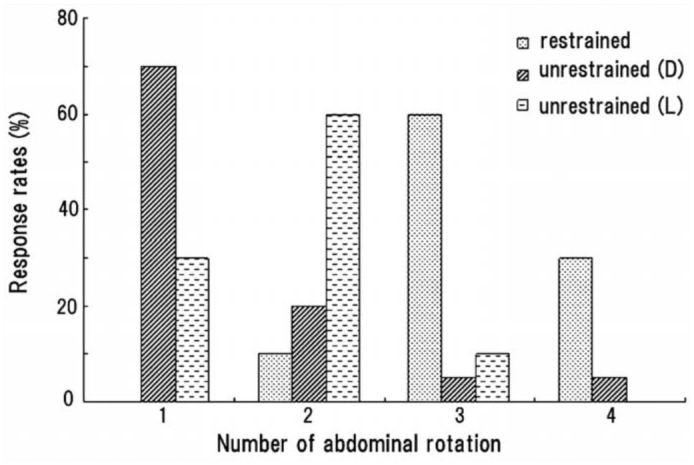
Mean ± SEM percentage of pupae responding to double brushing in relation to days after pupation. Soft (0.2 mN), weak (0.7 mN), and strong (1.7 mN) brushings were applied to the abdominal segment of six pupae 10 times per determination. High quality figures are available online.

Responsiveness to brushing the abdominal sternite changed to a great extent during the pupal period. The cuticle of a white pupa at 0– 3 hr after pupation was very soft, and a weak brushing (0.7 mN) on the soft cuticle of the abdomen usually induced a single rotation reflex rather than the sequential abdominal movements. When the brushing force was reduced to about 0.2 mN, the sequential abdominal movements were evoked by double brushing (or sometimes by single brushing), without the induction of a single rotation reflex. Effectiveness of the soft brushing rapidly decreased within the first four days of the pupal period, and double brushings with greater force were required to stimulate the pupa to perform the sequential movements with advances in development from a pupa to an adult (Figure 6). Pharate adults (13-day-old pupae) hardly exhibited any response to multiple brushings of the abdomen, although they could perform an abdominal rotation in response to tactile stimulation of the appendages.

**Figure 7.**
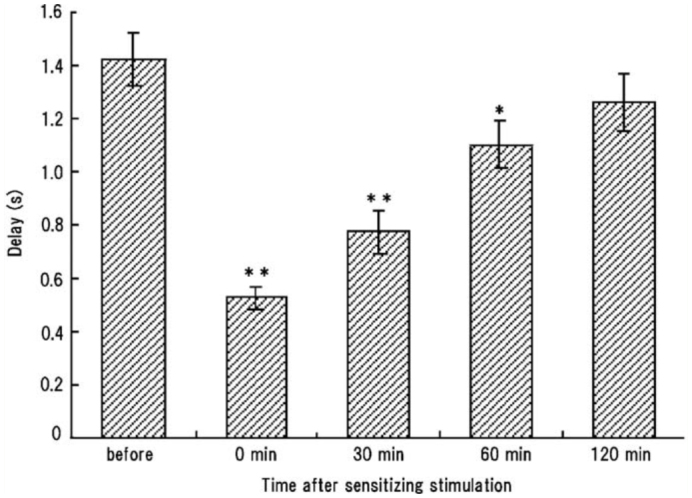
Decay of response sensitization caused by repeated (irritable) stimulation. Delay from single brushing to the initiation of a response was measured as a measure of sensitization before and after 20 prods to an elytron. Each bar indicates a mean value and standard error of the mean from nine pupae. Asterisks indicate that the delays after stimulation significantly differ from the delay before stimulation (**, *p* < 0.01; *, *p* < 0.05) (Dunnett test). High quality figures are available online.

To evaluate whether the defensive response to stimulation had any behavioral sensitization, responsiveness to single brushing was tested after the pupa was strongly sensitized by repetitive prodding of an appendage, resulting in an abdominal rotation reflex. The pupae could be activated to the maximum extent by 20 successive prods to an elytron at an interval of 1 sec. The mean ± SEM rate of responsiveness induced by a single brushing before such irritable stimulation was 25.8 ± 5.4% (n = 12), while the value was 61.7 ± 7.4% after the stimulation. Sensitization was associated with an acceleration of the response; the mean delay to the onset of the abdominal movements was significantly shortened from 1.42 sec before stimulation to 0.53 sec after stimulation (Figure 7). The mean number of rotations significantly increased from 2.1 ± 0.4 before stimulation to 3.9 ± 0.6 after stimulation (*p* < 0.01), and the mean number of all components also increased from 11.6 ± 0.8 to 15.2 ± 0.9 by the stimulation (*p* < 0.01, Dunnett test). The sensitization response appeared to last for approximately 1 hr (Figure 7).

Whether campaniform sensilla mediated the initiation of the response was tested. Since an elytron has no HS on its outer surface, campaniform sensilla may have mediated the response (Figure 4). However, the inner surface of an elytron could contact the outer surface of the hind wing bearing HS, if pressed (Kurauchi et al. 2011). To eliminate this contact, quick-drying glue was poured into the gap between the elytron and the hind wing. The treatment greatly reduced the mean ± SEM response rate from 38.6 ± 4.8% before the treatment to 6.0 ± 2.4% (n = 5) after the treatment (*p* < 0.01, Dunnett test).

### Unrestrained pupae

**Figure 8.**
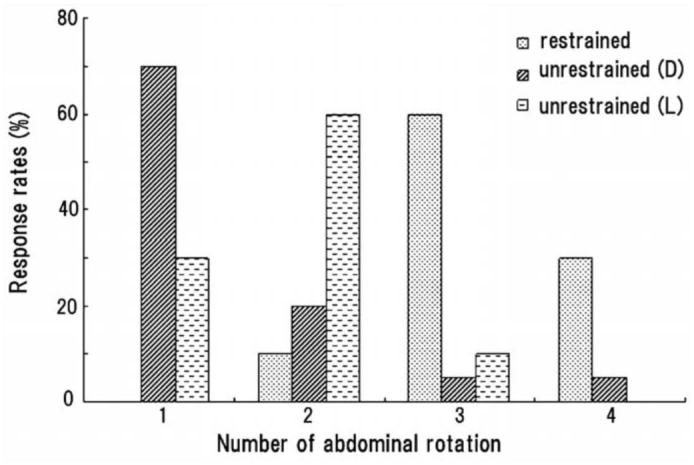
Abdominal rotations of a pupa in response to being restrained or unrestrained, positioned dorsally (D) or laterally (L) on the substrate. Twenty weak double brushings were applied to the abdominal segments under each condition. High quality figures are available online.

When an unrestrained pupa was laid on its dorsal or lateral side, a large abdominal movement (rotation or swing) usually caused the pupa to move away from the source of stimulation with the aid of the caudal processes (urogomphi), which served as a fulcrum to rotate the abdomen and thereby move the entire body. To examine the effect of such a displacement of the body on the sequential patterns of abdominal movements, an active pupa that usually rotated its abdomen 3–4 times under the restrained condition was laid on its dorsal or lateral side on a rough substrate. The pupa lying on its dorsal side usually reduced the number of sequential motions by inhibiting or skipping subsequent abdominal rotations after the displacement of its whole body caused by the first abdominal rotation (Figure 8). Mean ± SEM number of rotations (*N_r_*) and all components (*N_a_*) under the unrestrained condition were 1.4 ± 0.8 and 13.2 ± 2.4, respectively, while *N_r_* and *N_a_* under the restrained condition were 3.3 ± 0.6 and 16.2 ± 3.0, respectively. When the pupa was laid on its lateral side, it often exhibited an additional (2^nd^) rotation after the displacement of the body (Figure 8). *N_r_* and *N_a_* under the condition were 1.8 ± 0.6 and 13.3 ± 2.6, respectively. Similar results were obtained from six other pupae with moderate activity that usually exhibited two rotations under the restrained condition (data not shown).

## Discussion

*Z. atratus* pupae exhibit two types of reflex abdominal movements after mechanical stimulation of CS on the cuticle of the appendages and abdomen (Ichikawa et al. 2012a, b). The abdominal segments have many CS and HS. Since the CS can be fully activated by a weak force of less than 0.6 mN (Kurauchi et al. 2011), a weak brushing (0.6–0.7 mN) should activate CS as well as HS. The third defensive response of pupae failed to initiate after repeated prodding of the abdominal segment with the tip of a glass rod. Reduced stimulation of defensive responses was observed in an elytron having only CS (Figure 4) when masking HS on the outer surface of the hind wing beneath an elytron. This result suggested two possibilities: both types of mechanosensilla are necessary, or CS is unnecessary for initiating a response. Although the two possibilities could not be determined, the first possibility seemed likely because the rapid decline in sensitivity to soft brushing during the early pupal period (Figure 6) appeared to be closely related to a developmental increase in the stiffness of the pupal cuticle embedding the CS (Nakamura, unpublished observation). How many sensilla should be stimulated for the response? There are 5–20 HS/mm^2^ and 10–40 CS/mm^2^ on the surface of the abdominal segment (Kurauchi et al. 2011). The brushing area estimated by brushing the cuticular surface with a brush that had absorbed a small amount of paint was approximately 1 mm^2^. Thus, simultaneous activation of approximately 10 HS and 20 CS may be required for a response, although some HS may escape from contact with the brush.

The complex defensive movements of the abdomen in *Z. atratus* pupae consisted of variable sequential patterns and a relatively long delay to initiation of the movements after stimulation. This result suggested that the delayed response was not a simple reflex triggered by a sensory signal, and that there were complex neuronal mechanisms for the activation and execution of the sequential abdominal movements. Two successive brushings of an abdominal segment at an appropriate interval were usually needed to initiate the abdominal movements, and the effectiveness of the second brushing gradually decreased as the timing of the brushing was delayed (Figure 5). This result allowed us to propose a simple releasing mechanism for these movements on the basis of a synaptic facilitation mechanism, in which two temporally different postsynaptic potentials summate to exceed the threshold to generate an action potential. That is, this putative neuronal mechanism may have a slow and long-lasting process of excitation and a critical threshold for generating motor programs for the abdominal movements. A sensory signal generated by the first brushing may elevate the neural excitation level to a sub-threshold level, and the next signal induced by the second brushing further elevates the level to exceed the threshold. Brushing of other parts of the pupal body (Figure 4) may also contribute to the elevation of the excitation level of this putative neuronal mechanism. Other intense (irritable) stimuli, such as repetitive prodding of an appendage, might activate or sensitize the entire CNS rather than the neuronal mechanism alone, because a sensitized state lasted for 1 hr. This was apparently longer than the effective time of the first brushing (20–30 sec) (Figure 5), although the sensitization characteristics were not examined in detail in this study. Others have observed similar long-lasting sensitization of defensive responses by intense (noxious) mechanical stimulation in the larvae of *Manduca sexta* (Walters et al. 2001).

The sequential abdominal movements consist of the following three components: rotation, vibration, and wiggling. Trajectory patterns of the rotation were usually not simple circular or elliptical in shape, and they were characterized by an abrupt change in direction or a reversal of rotational direction halfway through the rotation (Figure 2). Ichikawa et al. (2012b) found that similar complex patterns of abdominal rotations were often induced by bilateral stimulations of appendages, while a unilateral stimulation of an appendage induced a clockwise or anticlockwise rotation of a simple elliptical shape. To account for this result, they proposed that the central nervous system might have two pattern generation systems, and the complex rotation patterns induced by the bilateral stimulations may be due to an interaction or interference between the two systems (Ichikawa et al. 2012b). Furthermore, each of the two putative systems for generating patterns seemed to consist of multiple pattern generators distributed in several abdominal ganglia of the abdominal segments (numbered A2–A6) having four bundles of intersegmental muscles (Ichikawa et al. 2012a). The complex trajectory patterns of abdominal rotations observed in this study (e.g., Figure 2) may be sums of clockwise and anticlockwise patterns of rotation made by activating the two sets of pattern generators at slightly different timings, even when the stimulation (brushing) was unilateral. Vibration and wiggling of the abdomen could represent cyclic lateral bends of all segments and several caudal segments, respectively. These two types of elementary motions may be produced by oscillatory pattern generators that alternately activate the right and left muscle bundles of the responsible segments.

The neuronal mechanism that executes the complex, sequential abdominal movements is largely unknown. However, a sensory feedback mechanism may exist for the control of motor programs of the movements, because a freely lying (unrestrained) pupa often does not perform the expected second and third rotations after the pupa changes the position of the body after the first rotation of the abdomen (Figure 8). Since longer HS are aligned along the lateral flanges of the prothoracic and abdominal segments, and CS are distributed in almost the entire pupal body in *Z. atratus* (Kurauchi et al. 2011), the sensilla may function as proprioceptors that monitor changes in position. These sensilla may also be involved in a feedback control mechanism of the putative pattern generators (Marder and Buchner 2001).

The abdomen of the insects generally has a mobile segmental structure connected by intersegmental membranes of the soft cuticle. The mobility of the abdomen is important for generating a periodic pumping activity that increases hydrostatic pressures in the hemocoel to facilitate circulation and respiration (Slárna 1988; Wasserthal 1996; Ichikawa 2008, 2009). However, such a mobile structure may increase the risk of attack by parasitoids and predators (Gross 1993). In this study, the sequential abdominal movements were most efficiently induced by repeated brushings on the ventral surface of an abdominal segment. These movements may be primarily effective in protecting the vulnerable abdomen from potential enemies, especially during the early pupal period (Figure 6). In the natural environment, crawling of a small enemy (such as a parasitoid wasp) on the ventral surface of the abdomen could induce an abdominal response, and the rapid and complex abdominal motions (vibrations and rotations) may function to dislodge the enemy. Although simple mechanical stimulation did not induce sequential abdominal movements, in contrast to the other two types of defensive responses, a variable pattern of abdominal movements lasted for a long period of time (Figure 2) if the movements occurred in response to a strong mechanical stimulation. In addition, repeated stimulation of the most vulnerable structures (appendages) of the pupal body (Figure 7) or rough handling facilitated a defensive response. These features of the pupal response suggest that the sequential abdominal movements may represent a desperate attempt at resisting repeated attacks from a large predator. The potential predators may include cannibalistic larvae of *Z. atratus* (Ichikawa and Kurauchi 2009).

A preliminary experiment revealed that a pupa of another species of tenebrionid beetle, *T. molitor*, also exhibited a sequence of abdominal movements consisting of several quick wiggling motions and swings of the abdomen. Although the start of the movements was significantly delayed 5–10 sec after the onset of a few prods or brushings to the soft pupal body, each induced an abdominal rotation. Strong stimulation sometimes could evoke a second or third sequence of movements intermittently during a period of 1–2 min after the stimulation. Thus, it is possible that the third defensive response is a common defensive behavior in pupae of many tenebrionid beetle species, although there may be a large variability in sensory and neuronal mechanisms and/or spatial and temporal patterns of the responses, depending on the morphology, physiology, and ecology of the beetles. Tenebrionidae is the most highly evolved and diverse family of Coleoptera, which is the largest order of living animals (Nielsen and Mound 2000). Comparative studies of the defensive mechanisms in pupae may provide valuable insights into the evolution and diversity of tenebrionid beetles.

## Abbreviations

**CS**,: campaniform sensilla
**HS**,: hair sensilla
